# Adaptation and Promotion of Emergency Medical Service Transportation for Climate Change

**DOI:** 10.1097/MD.0000000000000186

**Published:** 2014-12-12

**Authors:** Chih-Long Pan, Chun-Wen Chiu, Jet-Chau Wen

**Affiliations:** From the Graduate School of Engineering Science and Technology, National Yunlin University of Science & Technology, Douliou, Yunlin (C-LP); Department of Emergency Medicine, Changhua Christian Medical Center, Changhua (C-WC); and Department and Graduate School of Safety and Environment Engineering, Research Center for Soil & Water Resources and Natural Disaster Prevention, National Yunlin University of Science & Technology (J-CW), Douliou, Yunlin, Taiwan, ROC.

## Abstract

The purpose of this study is to find a proper prehospital transportation scenario planning of an emergency medical service (EMS) system for possible burdensome casualties resulting from extreme climate events.

This project focuses on one of the worst natural catastrophic events in Taiwan, the 88 Wind-caused Disasters, caused by the Typhoon Morakot; the case of the EMS transportation in the Xiaolin village is reviewed and analyzed. The sequential-conveyance method is designed to promote the efficiency of all the ambulance services related to transportation time and distance. Initially, a proposed mobile emergency medical center (MEMC) is constructed in a safe location near the area of the disaster. The ambulances are classified into 2 categories: the first-line ambulances, which reciprocate between the MEMC and the disaster area to save time and shorten the working distances and the second-line ambulances, which transfer patients in critical condition from the MEMC to the requested hospitals for further treatment.

According to the results, the sequential-conveyance method is more efficient than the conventional method for EMS transportation in a mass-casualty incident (MCI). This method improves the time efficiency by 52.15% and the distance efficiency by 56.02%. This case study concentrates on Xiaolin, a mountain village, which was heavily destroyed by a devastating mudslide during the Typhoon Morakot.

The sequential-conveyance method for the EMS transportation in this research is not only more advantageous but also more rational in adaptation to climate change. Therefore, the findings are also important to all the decision-making with respect to a promoted EMS transportation, especially in an MCI.

## Abstract 2

Supplemental Digital Content is available in the text

## INTRODUCTION

Over the last few decades, catastrophic effects of the climate change have drawn the attention of researchers worldwide.^1–4^ Diseases, injuries, and also deaths have resulted from storms, floods, droughts, fires, and heat waves, disasters which are on the increase.^2,4–8^ As a consequence, the large casualty caseloads led the emergency medical service (EMS) system to plan an adequate adaptation.^9,10^ Indeed, plethora of articles have been published to document the impact of the climate change on the EMS and the emergency medicine.^5,9,11,12^

During a mass-casualty incidence (MCI), the EMS transportation will be overwhelmed by the heavy caseload of casualties. Researchers and planners are concerned to shorten the referral and to save the golden hours.^13–17^ Plans, such as the 3 phases of prehospital patient care after an earthquake, are reviewed to reduce the immediate mortality.^18^ Moreover, other researchers apply the geographic information system (GIS) for a path design for effective time-saving transportation.^19^ In addition to all these, aerotransportation is another main force during an MCI, which is suggested to overcome the difficulties of the on-land transportation.^20^

How will the front-line workforce, the EMS, perform the duty of transportation effectively and save the golden hours for the casualties during a catastrophic event?^21^ Do the medical professionals arrive at the MCI and take care of casualties with nimble deftness?^22^ In this article, innovative ideas and plans for the EMS transportation and prehospital care are introduced, to abate the torment of sufferers from the tragedies caused by extreme climatic events.

A scenario of an effective EMS transportation, which is called the sequential-conveyance method in an MCI, is designed to shorten the transportation time and distance and to articulate with prehospital emergency care. Furthermore, an alternative concept of a mobile emergency medical center (MEMC) is introduced to integrate the EMS experts and the medics, so to act cooperatively and systematically. This MEMC is responsive to calamities and fully prepared to offer the emergency medical care close to the disaster area.

## METHODS

### Definitions

#### Ambulances

The definition of ambulances is general and it refers to both urgent and nonurgent ambulances. All types of ambulances are acceptable, but some calibrations of time used may be performed for certain types of ambulances.

#### EMS Adaptation

Because of climate change, which is almost unavoidable, EMS itself should concentrate on all the required dynamic changes to adjust to the new environmental and social conditions, which is required to act adequately in every situation.

#### First-, Second-, and Third-line Hospitals

In Taiwan, hospitals are basically classified into 3 different levels: the local community hospitals, the metropolitan hospitals, and the academic medical centers, depending on the evaluation of the quality and the quantity of the medical care. Typically, the services of the high-level hospitals are mostly related to serious health incidents, whereas low-level hospitals are for minor and nonurgent incidents. For this study, the EMS transportation for the local community hospitals is referred as first-line (1st-L H), for the metropolitan hospitals as second-line (2nd-L H), and for the academic medical centers as third-line hospitals (3rd-L H).

#### MEMC

What is proposed as a MEMC is a surge refuge, which is hybridized with a disaster-medical-aid center. The MEMCs have many differences compared with the traditional disaster-medical-aid centers or the casualty-collection points.^18,23,24^ To evacuate the area of an MCI, all the survivors, including the casualties, will be transferred to MEMCs, so that the required emergency medical treatment for injuries will be given. These MEMCs are the only intermediate facilities from the disaster area to the hospitals. Fully equipped trailers with medical personnel and necessities reach a specific location that must be safe and wide. Meanwhile, armamentaria and manpower are ready for patient care. Physicians in a MEMC can do further professional triage and assign transferrals of the second-line ambulances to the requested hospitals. This analysis mostly focuses on the aspects of the emergency transportation in MEMCs.

#### Patient Ratio

Survivors in a catastrophic event or an MCI are classified into 2 categories: the nonpatients (p) and the patients (q). Accordingly, the patient ratio (PR) can be presented by the following formula: PR = q/(p + q)

#### Sequential-Conveyance Method

**FIGURE 1:**
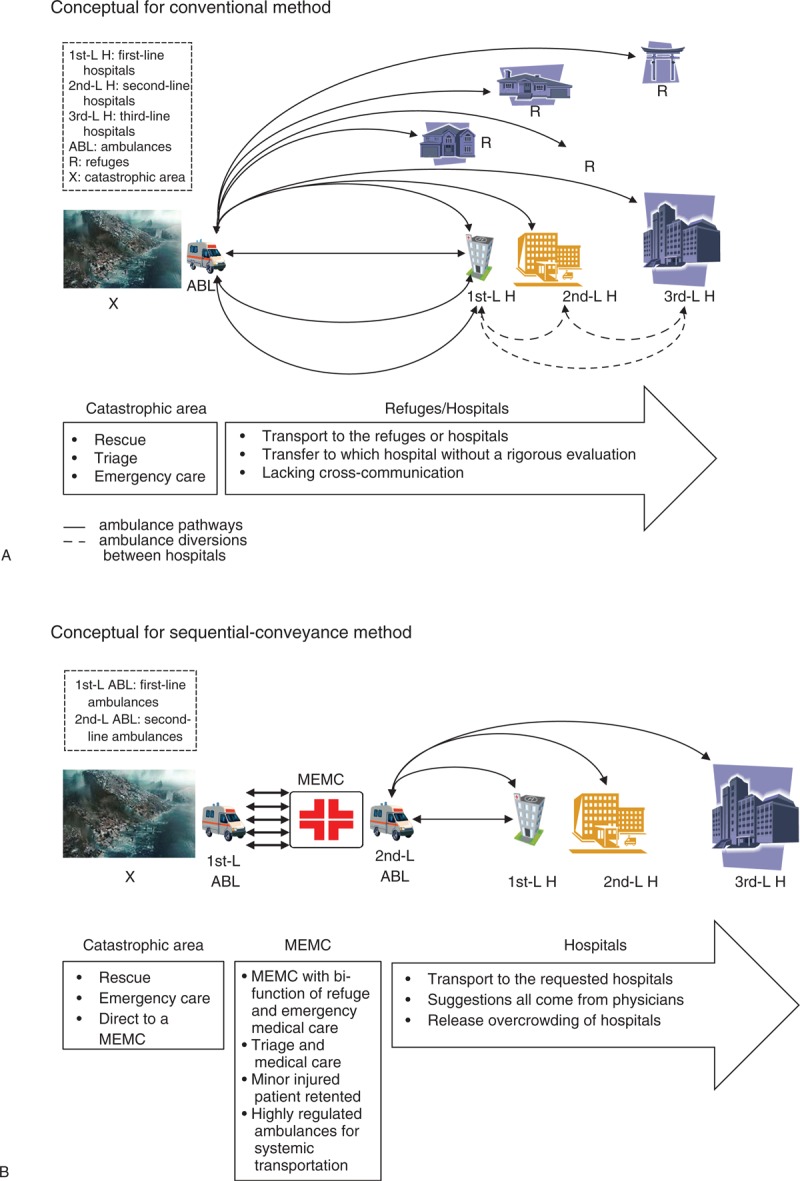
Diagram of the 2 different EMS transportation scenarios during an MCI. EMS = emergency medical service, MCI = mass-casualty incident.

For this EMS transportation, ambulances are categorized into 2 different lines. The first-line ambulances (1st-L ABL) transfer patients between the disaster area and a MEMC reciprocately. The benefits of this class are the saving of time and shortening of working distances. The second-line ambulances (2nd-L ABL) transfer patients between a MEMC and the requested hospitals, an action that directly transports heavily injured patients to specific hospitals only under the guidance of the physicians. A comparison between the conventional and the sequential-conveyance method is depicted in Figure 1.

#### Time/Distance Efficiency

To evaluate the efficiency of the EMS transportation between the conventional and the sequential-conveyance method, the difference of the required time and the difference of the distance of these 2 methods are estimated. The subtraction of the total EMS transportation time of the conventional (T_CM_) method from that of the sequential-conveyance method (T_SM_) divided by T_CM_ equals to TE. The subtraction of the total transportation distance of the conventional (D_CM_) method from that of the sequential-conveyance method (D_SM_) divided by D_CM_ equals to DE.

#### Total Transportation Time/Distance

The total transportation time is the required time for the action of an EMS transportation; it also includes the time destined for the incident area, the refuges, or the hospitals, the time for the triage, and the prehospital emergency care. Yet, the total transportation distance is the aggregate journey of the ambulances.

### Background Information

On August 8, 2009, Typhoon Morakot wrought havoc in Taiwan, by taking the lives of 702 individuals and by causing damage of roughly US$ 6.76 billion.^25^ Almost all the southern regions of Taiwan were flooded by a record-breaking heavy rain that caused damages beyond any imagination. This tragedy is also known as the “88 Wind-caused Disasters.”

The project concentrates on Xiaolin mountain village, which was heavily destroyed by a devastating mudslide during the Typhoon Morakot. It is believed that >400 residents buried alive, whereas 137 people were saved and sent to safety areas; 16 of them with injuries were transported to hospitals for further medical care.^26^

### A Scenario Setting

Two versions of casualty transportation are compared: the conventional and the sequential-conveyance method. Both are shown in Figure 1. In Panel A, the conventional method imitates the real scene of EMS transportation in Xiaolin village; whereas Panel B represents a virtual scenario of the sequential-conveyance method. In the conventional method, all casualties are sent to different lines of hospitals and as a result, anomalies of poor regulation and cross-communication may occur. This allows further diversions among hospitals (Figure 1: Panel A; dashed lines), which are performed frequently, and they are based on matters such as the emergency department (ED) crowding, the poor prehospital triage, and other problems similar to these.

The reason why Xiaolin village was selected for this study is its arduous casualty transportation. Jiasion elementary school, a safe and open space near the area of Xiaolin village, is chosen as an MEMC in the sequential-conveyance method. According to the records, all the casualties were transferred to 4 hospitals that can be classified into 3 lines. Chishan and Sijhou Hospital (denoted as 1st-L H), Tainan Municipal Hospital (denoted as 2nd-L H) and Chi Mei Medical Center (denoted as 3rd-L H) are assigned as first-line, second-line, and third-line hospital(s), respectively.

### Setting and Selection of Participants

Based on the official records,^26^ 137 people were found alive and rescued from the calamity-stricken village of Xiaolin during the Typhon Morakot; 16 of them were injured and sent to hospitals for further treatment. The original raw data of referrals are shown in Table 1. For the sequential-conveyance method, it is assumed that all the 135 people (the destination of 2 cases has not been registered) were sent to an MEMC and 16 of them were later sent to different lines of hospitals for further treatment. Two major different flow ratios are presented, one of the survivors who were transferred to hospitals and one of the survivors who were transferred to refuges (or a MEMC), to discriminate the patient flow and nonpatient flow between the 2 scenarios of conventional and sequential-conveyance methods. It is worth mentioning that an MEMC is a chimera of a refuge and a disaster-medical-aid center; therefore, all survivors were first sent to an MEMC and some of them were later transferred to hospitals for intensive care.

**TABLE 1:**
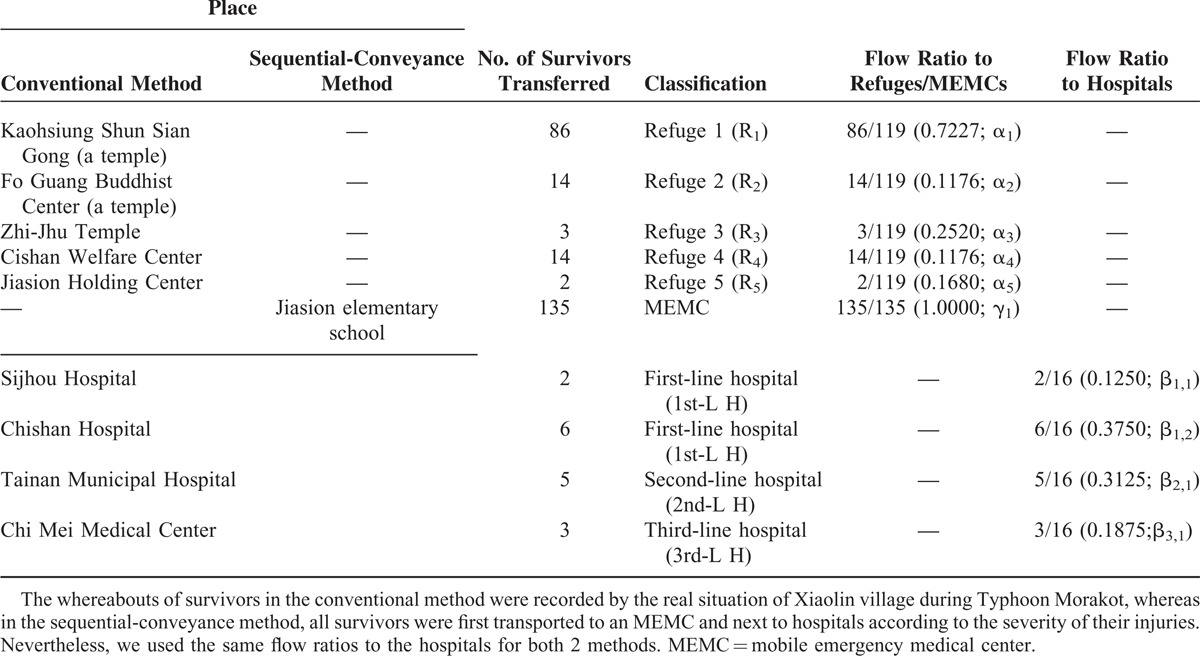
Whereabouts of Evacuated Survivors at Xiaolin Village During Typhoon Morakot in the Conventional and Sequential-Conveyance Method

### Measures

**FIGURE 2:**
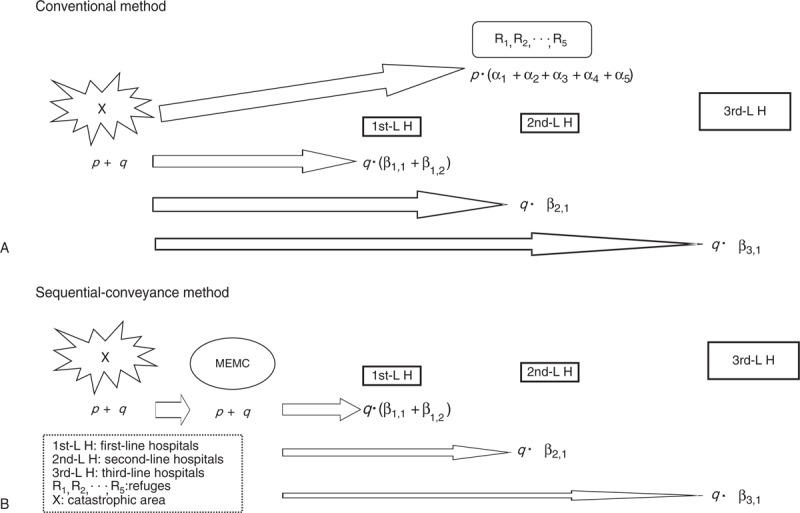
Patient and nonpatient flows in the 2 different transportation methods.

To estimate the efficiency of the sequential-conveyance method in the catastrophic events, Google Map^27^ has been chosen to simulate the transportation scenarios. The distance and the time interval between each dispatch are calculated by using the route-planning function of Google Map. In the case of Xiaolin, only 16 of 135 casualties in the MEMC were transferred to the different lines of hospitals, after assuming that the residuals were stabilized and readily treated in the MEMC. For a theoretical comparison between the conventional and the sequential-conveyance method, all assumptions are depicted in Figure 2. In Panel A, the survival flows in the conventional method are represented as survivals (p + q; p, nonpatients; q, patients) in the Xiaolin (X) event are sent to different destinations chaotically. Nonpatients are sent to refuges (R_1_–R_5_) and patients to hospitals (1st-L, 2nd-L, and 3rd-L H). Comparatively scenarioing, the sequential-conveyance method is indicated in Panel B.

### Data Analysis

A theoretical computation is performed to analyze the differences between the conventional and the sequential-conveyance method. As a matter of fact, the number of ambulances in situ is a limiting factor. Consequently, it is assumed that each transferral of an ambulance is an independent event and the time used can be accumulated. According to the concept of this study, casualties with minor injuries are gathered in an MEMC for the basic treatment. On the contrary, all the patients are sent directly to hospitals regardless the condition of the casualties in the conventional method. The T, D, TE, DE, TE versus PR, and DE versus PR are thoroughly deduced and equated in the Supplemental Content, http://links.lww.com/MD/A81 for further comparison.

## RESULTS

The intervals of distance and time of each dispatch are shown in Table 2 with the route-planning function of Google Map. For this project, 2 departure sites have been set: Xiaolin village and Jiasion elementary school (MEMC) but different destinations, ipso facto. Although the best suggested routes by Google Map (top-ranked routes) are usually preferred, sometimes different selections among the Google-suggested routes are more reasonable and efficient, due to factors such as the comparison between the time and the distance in real-life circumstances for such incidents in both methods. To minimize the discrepancy between the conventional and the sequential-conveyance method, the scenarios analyzed in this project are based on the following conditions: same destinations, same ratios of casualty distribution, and same road-routing program. A scenario of conventional method is shown in Figure 2: Panel A depicts the casualties being transported directly to different lines of hospitals without any programmed retention; Panel B depicts that the survivors of the sequential-conveyance method are first transferred to an MEMC for triaging, stabilizing, care-giving, and sheltering. Additionally, a programmatic and a subsequent transferral for the serious casualties is indicated by physicians in an MEMC.

**TABLE 2:**
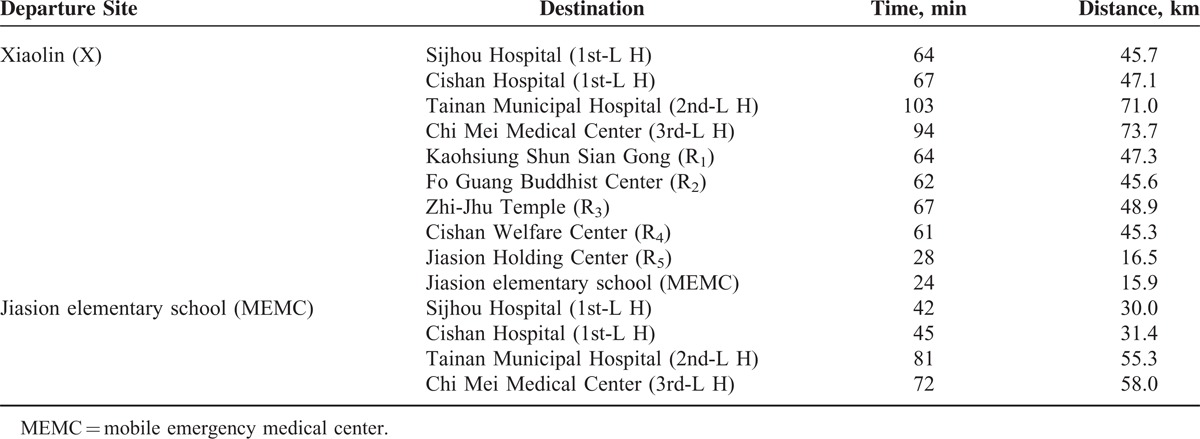
Distance and Time Interval to Each Dispatch of Ambulances Using the Route-Planning Function of Google Map

In the case study of Xiaolin village, values of TE and DE have been applied to evaluate the efficiency of EMS transportation time and distance, respectively. One hundred thirty-five people evacuated this event (p + q = 135) and 16 of them are sent to hospitals (q = 16). The ratios of the survivor flow to different destinations are shown in Table 1. Accordingly, the evaluation of the efficiency of the transportation time and distance in realistic conditions are TE = 0.5215 (52.15%) and DE = 0.5602 (56.02%) (see Eq. 10 and 17, Supplemental Content, http://links.lww.com/MD/A81, which show the deduction and computation processes analytically). These values show the predominance of the sequential-conveyance method over the conventional method in transportation time and distance of an EMS transportation.

**FIGURE 3:**
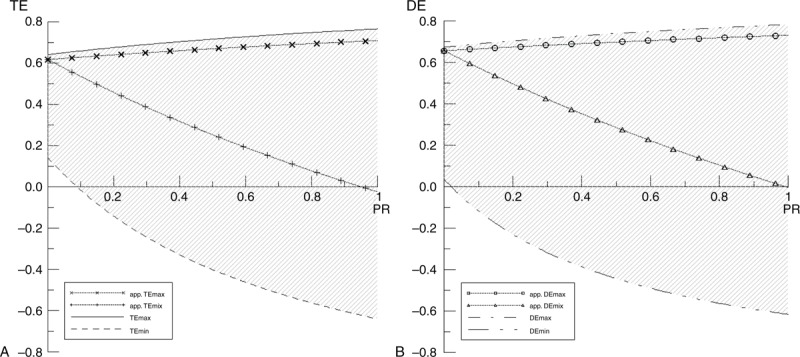
TE and DE (Panel A and B, respectively) analyses of the sequential-conveyance method compared with the conventional method by using different transportation maneuvers. DE = distance efficiency, TE = time efficiency.

The relations between the MEMC retention and the TE or the DE are depicted in Figure 3. In a concept of probability, each survivor would be transferred to each refuge or hospital equally and randomly. However, the PR in different supposed values would meet different T_CM_ and T_SM_ (see Eqs. 12 and 18, Supplemental Content, http://links.lww.com/MD/A81, which illustrate the connection between TE and PR, and between DE and PR, respectively) by permutation and combination algorithm in the conventional and the sequential-conveyance method. If a maximum T_CM_ encounters a minimum T_SM_ by a fixed PR, a maximum TE boundary (TEmax) (Figure 3: Panel A) can be obtained. On the contrary, when a minimum T_CM_ encounters a maximum T_SM_, a minimum TE (TEmin) is acquired. However, in a retrospective case study, the patient and the nonpatient flow of each hospital and each refuge, respectively, are kept in a determinate ratio (a fixed PR). In such situations, the maximum and minimum values of TE depend on the MEMC retention for the patients. Therefore, due to the MEMC retention (Figure 3: Panel A), an apparent TEmax (app. TEmax) and an apparent TEmin (app. TEmin) calculations are executed to evaluate the effect of the sequential-conveyance method. The same mathematical logic is applied for DEmax, DEmin, app. DEmax, and app. DEmin calculations (Figure 3: Panel B). In the real case of Xiaolin, if 16 patients (*PR* ≅ 0.1185) were subjected to the MEMC retention, the app. TEmax = 63.22% and app. DEmax = 66.88% would be obtained. Alternatively, since the patients are not retented to an MEMC, the app. TEmin = 52.15% and app. DEmin = 56.02% are obtained. These values are also equal to TE and DE, respectively.

## DISCUSSION

In an MCI, both EMS transportation time and distance have a high impact in a compressed window of emergency medical care when seconds and meters could mean life or death. Issues such as how to reduce the transportation time and distance will always be discussed for efficient solutions.^20,28–33^ In this research, a method of sequential-conveyance is conducted to promote the EMS transportation time and distance efficiency in 52.15% (app. TEmin) and 56.02% (app. DEmin), respectively, compared with the nonprogrammed conventional method. The findings also show that if all patients were retained in an MEMC, a value of app. TEmax and app. DEmax of 63.22% and 66.88%, respectively, would be obtained.

The app. TEmin and app. DEmin plots (Figure 3) show the efficiency of time and distance in the condition of no casualty retention in an MEMC. Patients in this situation are first transported to an MEMC with triage and first-aid treatments and then to different lines of hospitals at once. The process without any patient retention in an MEMC is considered as redundant, regardless the triage and the basic treatment by the medical personnel. A conservative MEMC capacity is determined by a positive or zero value of app. TEmin and app. DEmin, despite the contribution of app. TEmax and app. DEmax. For this case study, the selection of Jiasion elementary school as an MEMC could obtain a negative app. TEmin beyond 128 of patients (*PR* ≅ 0.9481). The lowest value of app. TEmin of the 135 of patients (PR = 1) is –2.41%. This is just a slight negative descent compared with the TEmin plot. The data show that the broad spectrum of the positive app. TEmin in the sequential-conveyance method can result in selecting a good location of the MEMC. In other words, when choosing the Jiasion elementary school as an MEMC, the minimum time efficiency can be kept under a capacity of 128 patients, even if all the casualties may eventually be removed to the requested hospitals without any retention in situ. The additional saved time can be used for triage and first-aid assistance to more patients. For the value of app. DEmin, the negative value begins at 135 of patients wherein –0.34% has been defined. It implies that the selected MEMC has distance ascendancy under a capacity of 134 patients.

In Panel A of Figure 3, the y-interceptions of app. TEmax and app. TEmin plots are on the same point (0.6183) because there is no patient available (PR = 0) for further transfer. It appears that the selected MEMC (the school) as a refuge has a time efficiency of 61.83%. Theoretically, the 135 nonpatient survivors are transferred to the 5 recorded refuges with a fixed flow ratio in the conventional method, whereas in the sequential-conveyance method, they are transported to the MEMC. In addition, the y-interception of app. DEmax and app. DEmin is 0.6572 (Figure 3: Panel B).

How to select an adequate MEMC by analyzing TE and DE? If the app. TEmax plot is closer to the TEmax plot and the app. TEmin plot has a positive shift from the TEmin plot, then these imply that the selected MEMC has time efficiency in the sequential-conveyance method, by keeping the advantage of the TEmax and by limiting the disadvantage of the TEmin. The same results are taken from a discussion either from the app. DEmax and the DEmax or the app. DEmin and the DEmin.

Aside all the positives, there is also a discussion about whether MEMCs’ bypass could cause an adverse effect for the patients who require immediate resuscitation. Some similar case studies regarding hospital bypassing to trauma centers may preclude the link between the bypass time and the survival in the study of Level I trauma patients.^34^ Moreover, patients with severe multiple traumas in the rural areas are suggested for triage and stabilization in Level III EDs before they are transferred to Level I regional trauma centers.^35^ In Taiwan, a previous study shows that there are no significant differences in mortality of severe trauma patients between a direct transportation to the study ED and a bypass transfer from another hospital after stabilization.^36^ Therefore, the establishment of MEMCs for patient triage, first-aid care, and stabilization before a long journey to hospitals are highly recommended in this research.

In Taiwan, patients are sent to the nearest hospitals depending on their own will or the judgment of their families, without any medical evaluation.^36^ This may lead to disturbance or overcrowding in the EDs, especially in an MCI and may cause an inadequate transferral or a redundant subsequent diversion. Given the benefits of MEMCs, all patients are diagnosed, treated, and clinical judged by the prehospital physicians therein; however, serious cases are suggested adequately to transfer to a requested hospital for intensive care. Controversies over the competencies and advantages for prehospital physicians are consistent with this scenario for MEMCs’ setting.^37,38^

The MEMCs should be set up as less as possible while a catastrophe occurs to avoid the silo-effect^39^ and a disordered cross-communication. Additionally, in Taiwan, the 3 phases of prehospital patient care may not be suitable for adoption due to the short-distance transportation of the patients and the lack of systemic guidelines.^40^

Under the issue of the climate change, a catastrophic calamity of an extreme weather event will force the EMS to do some adjustments for adaptation. It is difficult with a traditional method to manage a large caseload of casualty smoothly and methodically. That is why a systematic adaptation for EMS transportation is suggested because this strategy not only succeeds in efficient emergency transportation, but also highly regulates the distribution of patients to release the overcrowding of the responsible hospitals. Hence, additional practical work should be launched to collect more precise quantitative data.

## LIMITATIONS

Google Map provides a function of route-planning for optimal path suggestion by artificial intelligence, but sometimes the real situations of roads for ambulances’ progress are unpredictable, especially if extreme weather conditions occur. In fact, the judgment of the driver in decisions such as the road re-routing, the deceleration, the acceleration, or even the halt is very important in a calamitous transportation. Hence, the estimation of time and distance in this study will leave an uncertain erroneousness.

Moreover, this project tries to formulate a hypothesis to accumulate the time needed for each transportation. Although in an ideal scenario, the transfer units tend to correspond to the casualties, in real life, this probability is extremely low because the ambulances are limited in units.

Another limitation is the MEMC selection. Some potential locations have been designed in advance for such purposes, but they may not be suitable for sufficient operations in a realistic situation of an extreme weather condition. In other words, a theoretical TE or DE can be found by calculation and by using an expected MEMC, but in real-life conditions, the value of TE or DE can be relatively lower.

The time used in an MEMC for the first-aid treatment before transferring to hospitals is eliminated; however, this will create an error in time comparison between the conventional and the sequential-conveyance method when the retention time in an MEMC is long.

The EMS ground transportation is the major scheme for prehospital transferral in Taiwan. Nevertheless, some other disaster response actions may be operated, such as the aerotransportation when the roads are destroyed by weather phenomena such as the heavy rainfall, landslides, earthquakes, and other similar incidents. The concept of the sequential-conveyance method will not be affected by different EMS transportation vehicles because the MEMC will be treated as a relay station for ground and aerotransportation and the computation manners will be changed.
